# How effective is acupuncture in treating hot flashes in breast cancer patients? A systematic review and meta-analysis

**DOI:** 10.3389/fonc.2025.1543938

**Published:** 2025-03-17

**Authors:** Genlan Zhang, Cui Gao, Zining Guo, Wenrui Zhao, Xufang Xu, Huaneng Wen, Yaoxuan Li, Run Lin, Nenggui Xu, Shaoyang Cui

**Affiliations:** ^1^ Department of Rehabilitation Medicine, Shenzhen Hospital (Fu Tian) of Guangzhou University of Chinese Medicine, Shenzhen, China; ^2^ The Sixth Clinical Medical School, Guangzhou University of Chinese Medicine, Guangzhou, China; ^3^ South China Research Center for Acupuncture and Moxibustion, Medical College of Acu-Moxi and Rehabilitation, Guangzhou University of Chinese Medicine, Guangzhou, China; ^4^ Department of Rehabilitation Medicine, Shenzhen Hospital, Southern Medical University, Shenzhen, China

**Keywords:** acupuncture, hot flashes, breast cancer, systematic review, meta-analysis

## Abstract

**Background:**

Although acupuncture is recommended for managing breast cancer-related hot flashes, the level of evidence is limited. With the updating of randomized controlled trials, it is necessary to reassess its efficacy.

**Objective:**

To assess the effectiveness of acupuncture in the treatment of hot flashes in patients with breast cancer.

**Methods:**

Up to March 2024, we retrieved data from nine databases and used Stata software (version 14.0, version 17.0) and RevMan software (version 5.3) to conduct a meta-analysis. The Cochrane Collaboration’s risk of bias assessment tool was used for methodological assessment of the risk of bias, and the GRADEpro GDT online assessment tool was used for evidence evaluation.

**Results:**

In total, 11 randomized controlled trials (RCTs) involved 963 participants were included in the meta-analysis. The result of risk of bias revealed that the included RCTs exhibited a high risk of bias, primarily attributable to deficiencies in randomization and blinding methods. The results of primary meta-analysis indicated that acupuncture can improved the hot flash symptom scale score (SMD, -0.54; 95% CI, -0.83 to -0.24; *P <* 0.05). However, acupuncture does not reduce the frequency of hot flashes(SMD, -0.20; 95% CI, -0.75 to 0.36; *P = 0.48*). Further subgroup analyses, including the type of control group and the duration of needle retention, etc. showed different results, highlighting the necessity for further research. Sensitivity analysis confirmed the reliability of these finding. In addition, due to various issues, the level of evidence is low.

**Conclusions:**

Although acupuncture treatment for hot flashes in breast cancer shows potential, the evidence for the efficacy of acupuncture is still lacking due to various factors such as bias risk and significant differences between studies, and more high-quality RCTs are needed to confirm the efficacy of acupuncture.

**Systematic review registration:**

https://www.crd.york.ac.uk/prospero/, identifier CRD42024531542.

## 1 Introduction

The challenge posed by breast cancer to global public health remains severe, with an estimated 2.3 million new cases and 685,000 deaths each year (1). 80% of breast cancer patients experience hot flashes, which are characterized by a sudden onset of temporary fever with intense sweating (2, 3). Typically, mild hot flashes subside within minutes, while severe hot flashes persist for several hours. It is worth noting that persistent high intensity hot flashes will not only cause fatigue and sleep disorders, which will reduce the quality of life of patients, but may also affect patients’ adherence to anticancer treatment regimens, resulting in failure to achieve optimal therapeutic outcomes, and thus how to effectively manage hot flashes in breast cancer has become a major challenge worldwide (4). Currently, medication is the primary symptom management measure for hot flashes, and hormone replacement therapy (HRT) is the most widely used. Although HRT has achieved some positive results in improving hot flashes, there are side effects such as headache, nausea, palpitations and other uncomfortable symptoms, and even increase the risk of recurrence of hormone-receptor-positive breast cancer (5). In addition to hormone replacement therapy, non-hormonal drugs also have certain clinical applications, including antidepressants, such as venlafaxine and paroxetine; anticonvulsants, such as gabapentin and pregabalin; as well as other medications, such as clonidine and oxybutynin, etc. However, these medications also have a series of side effects, such as venlafaxine, which may cause dry mouth, decreased appetite, nausea, and constipation; oxybutynin may lead to difficulty in urination and cognitive impairment (6). Given the limitations of the aforementioned drug therapies, neurokinin receptor antagonists are receiving increasing attention due to research on novel regulatory pathways for reproductive function. Neurokinin-3 receptor antagonists, represented by fexofoliratan, which was approved by the U.S. Food and Drug Administration (FDA) in 2023, have been proven to be more effective than traditional non-hormonal drugs in reducing the frequency of moderate to severe hot flashes (5). Elinzanetant is an antagonist that can simultaneously target NK1 and NK3 receptors after fexofoliratan, and it has also shown encouraging results in phase III clinical trials, significantly reducing the frequency and severity of moderate to severe vasomotor symptoms, improving sleep disturbances, and enhancing overall quality of life (7–9). Although emerging neurokinin receptor antagonists have shown some potential, the lack of sufficient clinical trials to support the quality of evidence means that further research is still needed to prove their exact efficacy and safety. Therefore, there is an urgent need for a complementary alternative therapy with higher safety and effectiveness to participate in the management of hot flashes in breast cancer patients.

In recent years, complementary and alternative therapies have gained significant momentum in the field of oncology. According to data from the National Health Interview Survey (NHIS) in the United States, more than one-third of cancer survivors have used complementary and alternative therapies within a year. Notably, breast cancer patients are the most frequent users (10, 11). Acupuncture, as one of the important interventions in complementary and alternative therapies, has garnered attention for its effectiveness in alleviating hot flashes in breast cancer patients, thanks to its high safety and ease of operation (10, 12, 13). The latest American Society of Clinical Oncology (ASCO) research indicates that acupuncture can improve the condition of breast cancer patients experiencing hot flashes in the United States, South Korea, and China (14). Moreover, China’s evidence-based guidelines on hot flashes have already recommended the use of acupuncture for treating hot flashes in breast cancer patients, albeit as a weak recommendation (evidence level C) (15). The Society for Integrative Oncology (SIO) in the United States also suggests that acupuncture can be considered to reduce the frequency of hot flashes in breast cancer patients, based on patient preference (16). It is noteworthy that the Oncology Nursing Society guidelines only recommend considering the use of acupuncture for hot flashes in breast cancer within the context of clinical trials (17). Given the differing perspectives on acupuncture for hot flashes in breast cancer patients and the recent emergence of evidence on this topic, it is necessary to conduct a comprehensive systematic review and meta-analysis to evaluate and summarize the evidence on the effectiveness of acupuncture in improving hot flashes in breast cancer patients, as well as to identify gaps and future trends in this field.

## 2 Methods

### 2.1 Protocol and registration

This review was conducted in accordance with the Preferred Reporting Items for Systematic Evaluation and Meta-Analyses guidelines. The review protocol is registered in PROSPERO (no. CRD42024531542).

### 2.2 Search strategy

Two reviewers (GZ and CG) independently conducted a comprehensive search of the studies in nine electronic databases (PubMed, Web of Science, Sinomed, Embase, Cochrane Library, EBSCO, China National Knowledge Infrastructure, China Science and Technology Journal Database [VIP], and Wanfang Database) from database inception to March 2024. The searches were conducted using medical subject heading (MeSH) terms and free words, with MeSH terms including “Breast Neoplasm (MeSH),” “Acupuncture (MeSH),” “hot flashes (MeSH),” “Breast Tumors,” “breast cancer,” “acupuncture therapy,” “acupuncture, ear,” “hot flush,” “hectic fever,” and “randomized controlled trial.” The reference lists of the retrieved articles were manually searched and previously published review articles were scrutinized. The search process for the Embase database is detailed in the following flowchart (**Table 1**).

**Table 1:** The retrieval process of the Embase database.

No.	Query	Results
#11	#5 AND #8 AND #9 AND #10	112
#10	‘randomized controlled trial’:ab,kw,ti OR ‘randomized’:ab,kw,ti OR ‘placebo’:ab,ti,kw	1184233
#9	#1 OR #2	761302
#8	#6 OR #7	20773
#7	‘flashes, hot’:ab,kw,ti OR ‘hectic fever’:ab,kw,ti OR ‘hot flushes’:ab,kw,ti OR ‘tidal fever’:ab,kw,ti OR ‘flush, hot’:ab,kw,ti OR ‘thot flash’:ab,kw,ti OR ‘hot flashes’:ab,kw,ti OR ‘hot flushing’:ab,kw,ti OR ‘hot flush’:ab,kw,ti	8543
#6	‘hot flush’/exp	19248
#5	#3 OR #4	66299
#4	‘acupuncture’/exp	59260
#3	‘acupuncture’:ab,kw,ti OR ‘acupuncture therapy’:ab,kw,ti OR ‘acupuncture, ear’:ab,kw,ti OR ‘electroacupuncture’:ab,kw,ti OR ‘acupuncture points’:ab,kw,ti OR ‘electro-acupuncture’:ab,kw,ti OR ‘needling’:ab,kw,ti OR ‘scalp acupuncture’:ab,kw,ti OR ‘pharmacopuncture’:ab,kw,ti OR ‘acupuncture treatment’:ab,kw,ti OR ‘acupuncture treatments’:ab,kw,ti OR ‘treatment, acupuncture’:ab,kw,ti OR ‘therapy, acupuncture’:ab,kw,ti OR ‘pharmacoacupuncture treatment’:ab,kw,ti OR ‘treatment, pharmacoacupuncture’:ab,kw,ti OR ‘pharmacoacupuncture therapy’:ab,kw,ti OR ‘therapy, pharmacoacupuncture’:ab,kw,ti OR ‘acupotomy’:ab,kw,ti OR ‘acupotomies’:ab,kw,ti OR ‘auricular acupuncture’:ab,kw,ti OR ‘acupuncture, auricular’:ab,kw,ti OR ‘acupunctures, auricular’:ab,kw,ti OR ‘acupoints’:ab,kw,ti OR ‘acupoint’:ab,kw,ti OR ‘point, acupuncture’:ab,kw,ti OR ‘points, acupuncture’:ab,kw,ti OR ‘acupuncture point’:ab,kw,ti OR ‘auricular acupunctures’:ab,kw,ti	50453
#2	‘breast neoplasm’:ab,kw,ti OR ‘neoplasm, breast’:ab,kw,ti OR ‘breast tumors’:ab,kw,ti OR ‘breast tumor’:ab,kw,ti OR ‘tumor, breast’:ab,kw,ti OR ‘tumors, breast’:ab,kw,ti OR ‘neoplasms, breast’:ab,kw,ti OR ‘breast cancer’:ab,kw,ti OR ‘cancer, breast’:ab,kw,ti OR ‘mammary cancer’:ab,kw,ti OR ‘cancer, mammary’:ab,kw,ti OR ‘cancers, mammary’:ab,kw,ti OR ‘mammary cancers’:ab,kw,ti OR ‘malignant neoplasm of breast’:ab,kw,ti OR ‘breast malignant neoplasm’:ab,kw,ti OR ‘breast malignant neoplasms’:ab,kw,ti OR ‘malignant tumor of breast’:ab,kw,ti OR ‘breast malignant tumor’:ab,kw,ti OR ‘breast malignant tumors’:ab,kw,ti OR ‘cancer of breast’:ab,kw,ti OR ‘cancer of the breast’:ab,kw,ti OR ‘mammary carcinoma, human’:ab,kw,ti OR ‘carcinoma, human mammary’:ab,kw,ti OR ‘carcinomas, human mammary’:ab,kw,ti OR ‘human mammary carcinomas’:ab,kw,ti OR ‘mammary carcinomas, human’:ab,kw,ti OR ‘human mammary carcinoma’:ab,kw,ti OR ‘mammary neoplasms, human’:ab,kw,ti OR ‘human mammary neoplasm’:ab,kw,ti OR ‘human mammary neoplasms’:ab,kw,ti OR ‘neoplasm, human mammary’:ab,kw,ti OR ‘neoplasms, human mammary’:ab,kw,ti OR ‘mammary neoplasm, human’:ab,kw,ti OR ‘breast carcinoma’:ab,kw,ti OR ‘breast carcinomas’:ab,kw,ti OR ‘carcinoma, breast’:ab,kw,ti OR ‘carcinomas, breast’:ab,ti	554302
#1	‘breast tumor’/exp	699373

### 2.3 Inclusion criteria

The inclusion criteria were as follows: (a) Patients with pathologically diagnosed breast cancer who experienced hot flashes (b) Intervention: The experimental group was limited to acupuncture as the sole intervention measure (unrestricted factors included the needle material, treatment point selection, manipulation techniques, and needle retention time), including electroacupuncture, fire needle, and ear acupuncture. The control group received sham acupuncture, conventional medication, no treatment, or were placed on a waiting list (c) Outcome: This study focused on hot flash symptoms in patients with breast cancer, examining the frequency of hot flashes (times/24 h, times/day, times/night), hot flash symptom scores (hot flash score, HFS), hot flash composite score, hot flash-related daily interference scale, Kupperman’s index (KI), visual analog scale, and other recognized effective scales. (d) Study type: Randomized controlled trial, with no restrictions on language.

### 2.4 Exclusion criteria

The exclusion criteria were as follows: (a) The experimental group used acupuncture therapy in combination with other therapies. Additionally, studies were excluded if they did not mention skin penetration during acupuncture, such as laser acupuncture, and if the control group used another acupuncture method. (b) Studies that did not focus on improving hot flash symptoms in postoperative patients with breast cancer as a main research indicator. (c) Animal experiments. (d) Duplicate publications. (e) If only the abstract was available and the full text was not obtainable.

### 2.5 Study selection

The retrieved literature was imported into NoteExpress. After deduplication, two investigators (GZ and XX) independently read the titles and abstracts of the literature according to the inclusion and exclusion criteria, performed the initial screening according to the inclusion criteria, and subsequently downloaded and read the full texts of the selected literature to make the final inclusion decision. For studies with incomplete original data, attempts were made to contact the original authors via email or telephone to acquire the necessary information.

### 2.6 Data extraction

Two investigators (GZ and CG) independently extracted data from the included original studies and created a database using Microsoft Excel. Information such as the source of the literature, stage of the tumor, intervention measures and duration, measurement indicators, results (mean and standard deviation), and methodological aspects including concealed randomization, intention-to-treat analysis, and blinding were extracted. Any discrepancies related to study inclusion or data extraction were resolved by a third researcher (ZG).

### 2.7 Risk of bias assessment

The two reviewers (GZ and CG) independently applied the Cochrane Risk of Bias Tool version 1 to assess the risk of bias in randomized trials. This tool encompasses six domains: (a) sequence generation and allocation concealment; (b) blinding of participants and outcome assessment; (c) blinding of outcome assessors (detection bias); (d) incomplete outcome data; (e) selective outcome reporting; (f) other biases. Each bias risk assessment was categorized as high, low, or unclear (18). In the event of discrepancies, a third researcher (ZG) made a final decision.

### 2.8 Quality of evidence

Two reviewers (GZ and CG) evaluated the quality of the evidence using the Grading of Recommendations Assessment, Development and Evaluation approach within GRADEpro GDT (http://guidelinedevelopment.org/). The evidence was assessed and classified as high, moderate, low, or very low. The assessment included the risk of bias, inconsistency, indirectness, imprecision, and other considerations. Disagreements were resolved through discussion and consultation with a third reviewer (ZG).

### 2.9 Statistical analyses

Stata software (versions 14.0 and 17.0) and RevMan 5.3 software were used to conduct the meta-analysis. Continuous variables measured in identical units were analyzed using the mean difference (MD) ± standard deviation (SD) as the effect size, while those measured in different units were analyzed using the standardized mean difference (SMD) ± standard deviation. The 95% confidence interval (CI) was computed, with a *P*-value < 0.05 denoting statistical significance. Heterogeneity among the studies was then evaluated. The Cochrane *I²* was used as a criterion for heterogeneity, and the I² was used for reaction heterogeneity statistic. If *P* ≥ 0.1 and *I^2^* < 50%, the studies were considered to exhibit homogeneity, and a fixed-effects model was applied for the meta-analysis. Conversely, if *P* < 0.1 and *I^2^* ≥ 50%, heterogeneity was identified, and a random-effects model was adopted. Subgroup analyses were performed to investigate the potential origins of heterogeneity. For studies numbering at least 10, a funnel plot and Egger’s test for asymmetry were used to evaluate publication bias. The quality and certainty of the evidence were tabulated.

## 3 Results

### 3.1 Study selection

**Figure 1:**
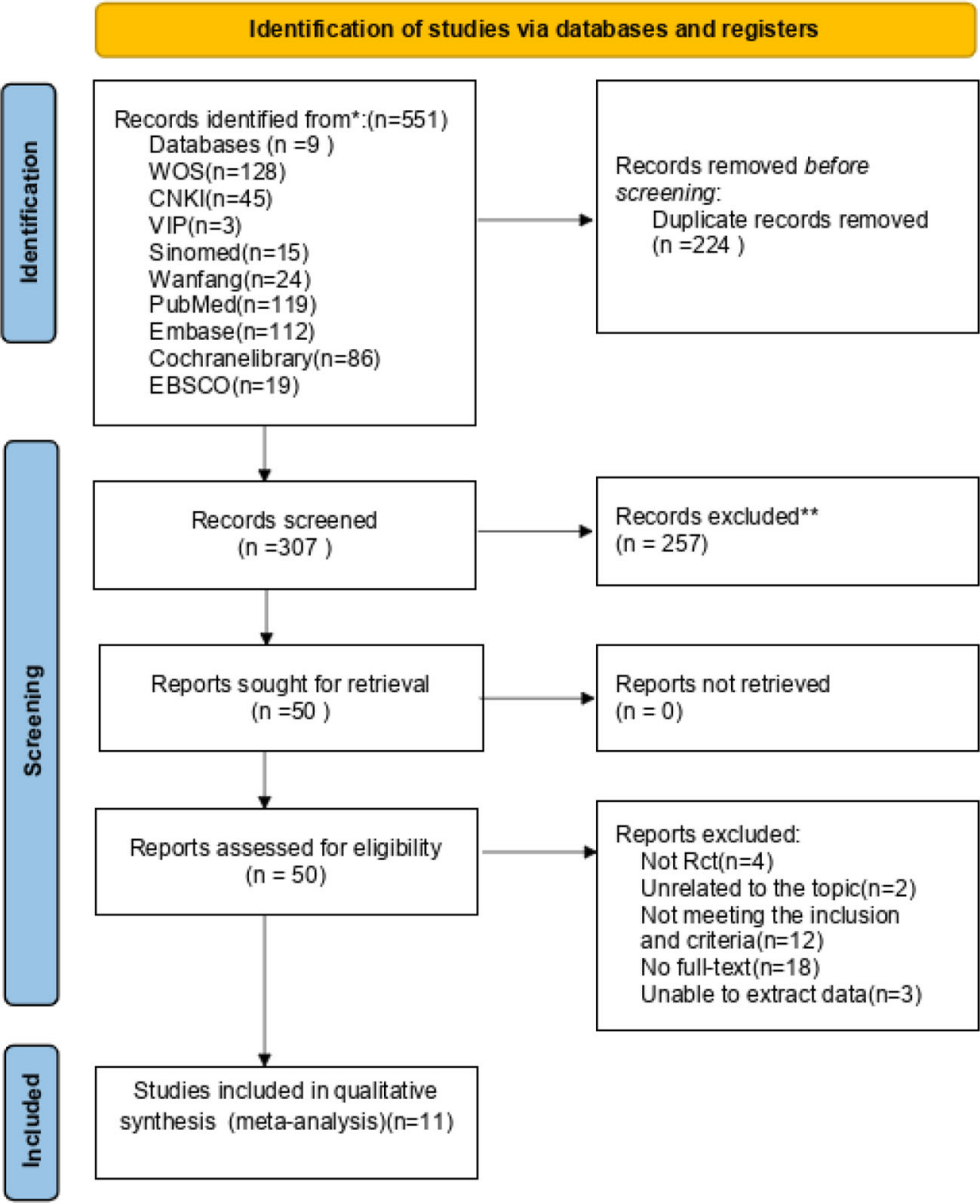
Flow diagram of screening process.

The initial search identified 551 articles, of which 307 were removed as duplicates. An additional 257 articles were excluded following a review of their titles and abstracts owing to irrelevance. Fifty articles were deemed suitable for inclusion. After assessing the full texts, 36 articles were excluded. Finally, 14 articles were included in the systematic review. However, two articles did not provide the standard deviation, precluding the extraction of complete data (19, 20). One article, presented only in a graphical format without specific data in the text (21), lacked the necessary information for meta-analysis and was omitted from the analysis after unsuccessful attempts to contact the authors via email. Finally, 11 articles were included in the meta-analysis (**Figure 1**). In the process of preparing for submission, we meticulously selected an academic article that aligned with the predefined inclusion and exclusion criteria, offering a valuable academic reference for the nuanced interpretation of the study outcomes (14).

### 3.2 Study characteristics

The 14 studies involved 963 participants, with 490 in the intervention group and 473 in the control group, and were published between 2006 and 2024. Five studies were conducted (19–23) in the United States, one in Denmark (19), two in Norway (24, 25), one in South Korea (26), one in China (14) and four in Sweden (27–30). Six studies used sham acupuncture as the control (22–25, 28, 31), two used medication as the control (20, 30), two used a blank control (22), one study chose application relaxation as the control group (27) and one study selected delayed acupuncture as the control group (14). The primary outcome measure in four studies (14, 20, 22, 32) was the HFS score; in four studies (24, 25, 27, 30) it was the KI score; and in five studies (23, 24, 27–29) it was the frequency of hot flashes. The duration of treatment and follow-up ranged from two weeks to three years. **Table 2** presents the general characteristics of the included studies. The results of the literature quality assessment are presented below. Finally, a total of 11 studies were included in this meta-analysis.

**Table 2:** Study characteristic.

Author/Year	Country	Sample	Age (Intervention/Control.years)	Intervention	Control	Cancer stage	Acupuncture points	Outcome	Acupuncture treatment and follow-up time	Needle retention time and Needle insertion depth	Advent	Registration Info
Nedstrand(2006) (27)	Sweden	38	53	Electroacupuncture	Applied relaxation	No mention	BL23,BL32	1.hot flushes/24 hours 2.Kupperman Index 3.VAS	Twice a week for the first two weeks and once a week for another 10 weeks follow-up was six months	30 min and 5-20 mm	no	no clear
Deng (2007) (23)	New York	72	55 (48–59)/56 (49 –59)	Acupuncture	Sham acupuncture	No mention	no clear	Hot flash frequency	Twice a week for 4 weeks followed up for 6 months	20 min and 6.35-12.7 mm	Only very minor adverse effects, such as slight bleeding or bruising at the needle site	no clear
Frisk (2008) (30)	Sweden	41	56.5/53.4	Electroacupuncture	Hormone therapy	No mention	no clear	1.Hot flushes/24 h 2.Kupperman’s Index	Twice a week for the first 2 weeks and once a week for the next 10 weeks followed up for 24 months	30 min and no mention	no	no clear
Jill Hervik (2009) (24)	Norway	59	53.6 ± 6.4/52.3 ± 6.9	Acupuncture	Sham acupuncture	No mention	DU14,GB20,BL13, PC7,H6,K7,ST36,SP6, Ear shen men,Ear sympathetic point	1.Kupperman (KI) 2.Hot flashes at day and night	The first 5 weeks, 2 times a week, after 5 weeks, 1 times a week followed up for 3 months	30 min and 5-30 mm	no mention	no clear
Walker (2010) (21)	American	50	52.2± 10.29/56.6± 8.27	Acupuncture	Venlafaxine	0-III	GV16, GV20, BL23,CV6	Hot flash frequency	Twice a week for the first 4 weeks and once a week for the next 8 weeks follow-up for 12 months	20 min and 6.35-12.7 mm	no	no clear
Frisk (2011) (29)	Sweden	45	54.1 (47–69)/53.4 (43–67 )	Electroacupuncture	Hormone therapy	No mention	no clear	1.The HFS 2. Hot flushes	The course of treatment was 12 weeks follow-up was 2 years	no mention	no	no clear
Liljegren (2012) (28)	Sweden	84	58 ± 6.8/58 ± 9.3	Acupuncture	Sham acupuncture	No mention	GV20,EX-HN3, HT8,KI10,LV2	1.Frequency of hot flushes and sweating per 24h 2.The severity of hot flushes and sweating per 24h	Twice a week for 5 weeks followed up for 18 weeks	20 min and 5-20 mm	no	no clear
Bokmand (2012) (19)	Denmark	94	60 (46–75)/62 (43–72)/62 (45–75)	Acupuncture	Sham acupuncture/ No treatment	No mention	urinary bladder 23, kidney 3, spleen 6, du 14, gallbladder 20, lung 9, liver 3, du20, stomach 36, ren6, pericardium7 and heart 7	VAS	Once a week for 5 weeks followed up for 12 weeks	15-20 min and no mention	fatigue, pruritus, and nausea	yes
Ting (2014) (31)	American	47	61(45-85)/61(44-82)	Acupuncture	Sham acupuncture	0-III	CV4,CV6, CV12, LI4, MH6, GB34,ST36,KI3, BL65,14 sham acupoints located at the midpoint of the line connecting 2 real acupoints.	1.HADS 2.the Hot flash daily diary 3.HFRDI	Once a week for 8 weeks followed up for 12 weeks	no mention	no mention	yes
Hervik (2014) (25)	Norway	88	52.5/50.2	Acupuncture	Sham acupuncture	No mention	Li4,Ht6, LR3,St36, Sp6,Ki7	Kupperman Index	A total of 15 treatments were performed for 10 weeks followed up for 2 years	no mention	no	no clear
Mao (2015) (20)	American	120	52.9 ± 8.6/52 ± 8.9/52.6 ± 8.2/50.4 ± 8.4	Electroacupuncture/Sham acupuncture	Gabapentin/ Placebo	I-III	LIV3,GB20,LU7,KI3, SP6,REN4,P7,LIV8	HFCS	Twice a week for 2 weeks, once a week for 6 weeks followed up for 24 weeks	30 min and no mention	no	yes
Serra, D (2023) (22)	New York	47	51 (28–69)/55 (41 –72)	Acupuncture	Sham acupuncture	I-III	Hc6,Ki3,Sp6,Lr3	HF	Twice a week for 5 weeks then once a week for 4 weeks followed up for 1 month	30 min and no mention	no	no clear
Jeong (2023) (32)	Korea	30	48.7 ± 4.10/46.2 ± 6.9	Acupuncture	No treatment	No mention	no clear	1.VAS 2.Total hot flash score	Three times a week for 4 weeks, followed up for 8 weeks	25 min and 5-10 mm	no	yes
Lu,W (2024) (14)	China	158	48 (31–72)/48 (25–73)	Immediate acupuncture	Delayed acupuncture control	0–III	SP‐6, LI‐11, Yintang, GV‐20, Shenmen/ear,LR3, ST36, K3, PC7, CV6, and Heart/ear	HFS	Treatment for 10 weeks, twice a week follow-up was 20 weeks	30 min and no mention	no	yes

### 3.3 Risk of bias

**Figure 2:**
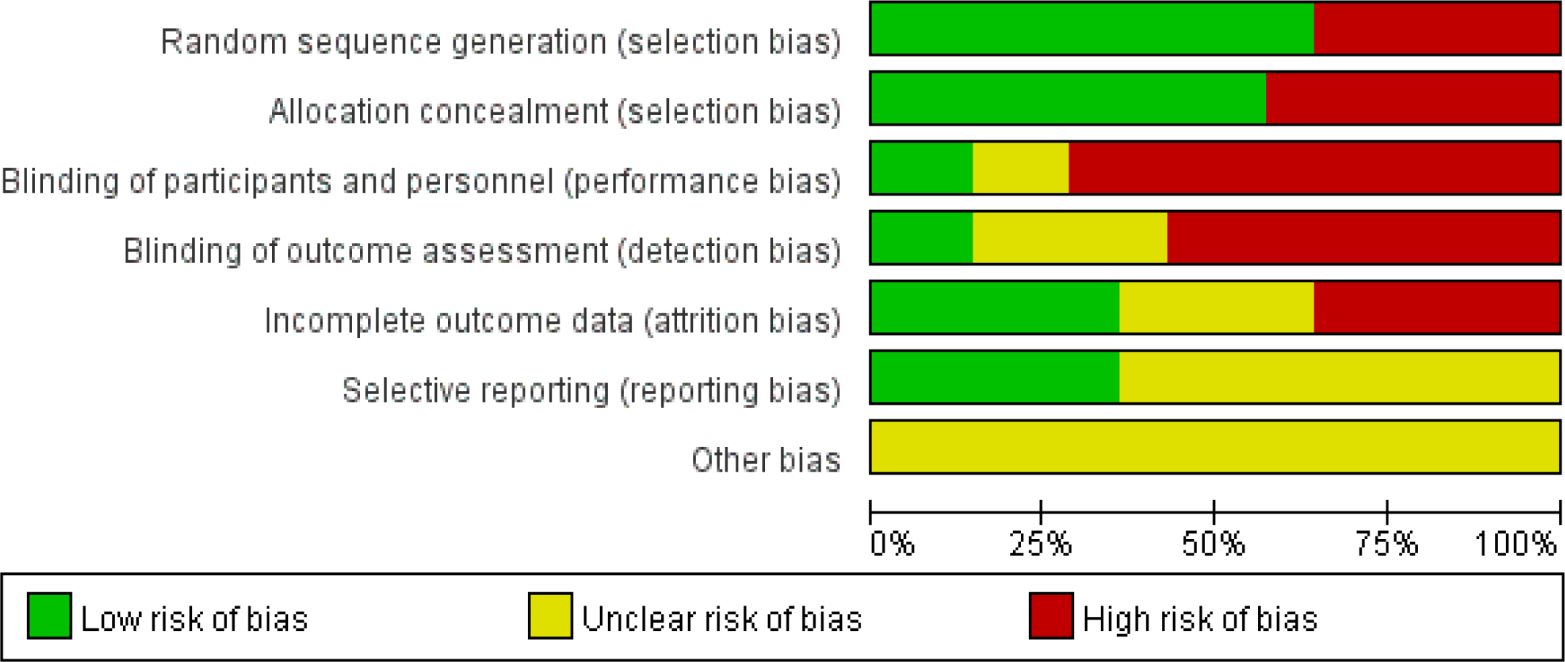
Risk of bias.

**Figure 3:**
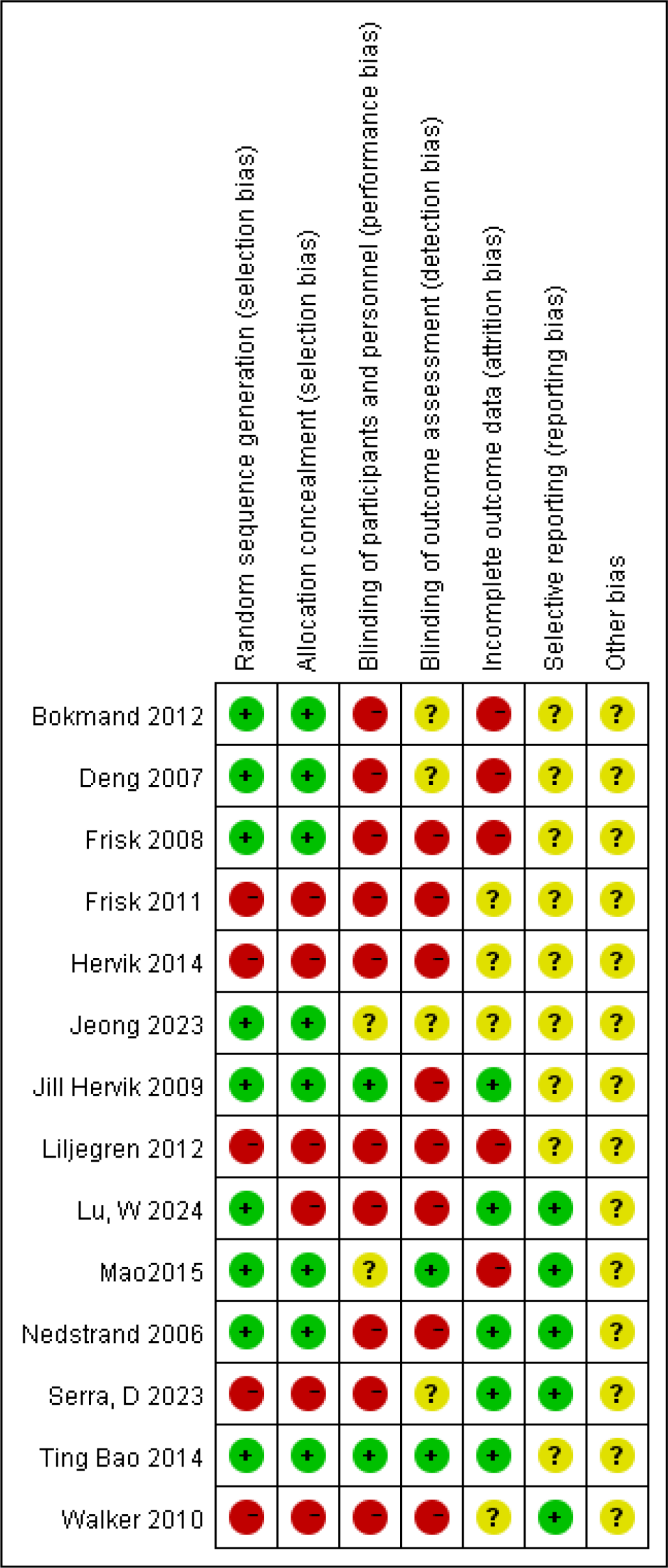
Risk of bias.

**Figures 2**, **3** indicate that for random sequence generation, nine studies mentioned the use of computer-generated random tables or sealed envelopes, which were considered to have a low risk of bias, whereas five studies did not describe the relevant content and were considered to be at high risk. Regarding allocation concealment, six studies were considered to have a high risk of bias. For blinding, ten studies were considered to have a high risk of bias in terms of blinding the participants and outcome assessment, and only two studies evaluated the blinding of outcome assessment, which was considered to have a low risk of bias. For incomplete outcome data, five studies did not mention the number of missing participants or reasons and were considered to be at high risk. Overall, the studies included were unsatisfactory in terms of random sequence generation, allocation concealment, participant blinding, and incomplete outcome data.

### 3.4 Meta-analysis of the frequency of hot flashes

**Figure 4:**
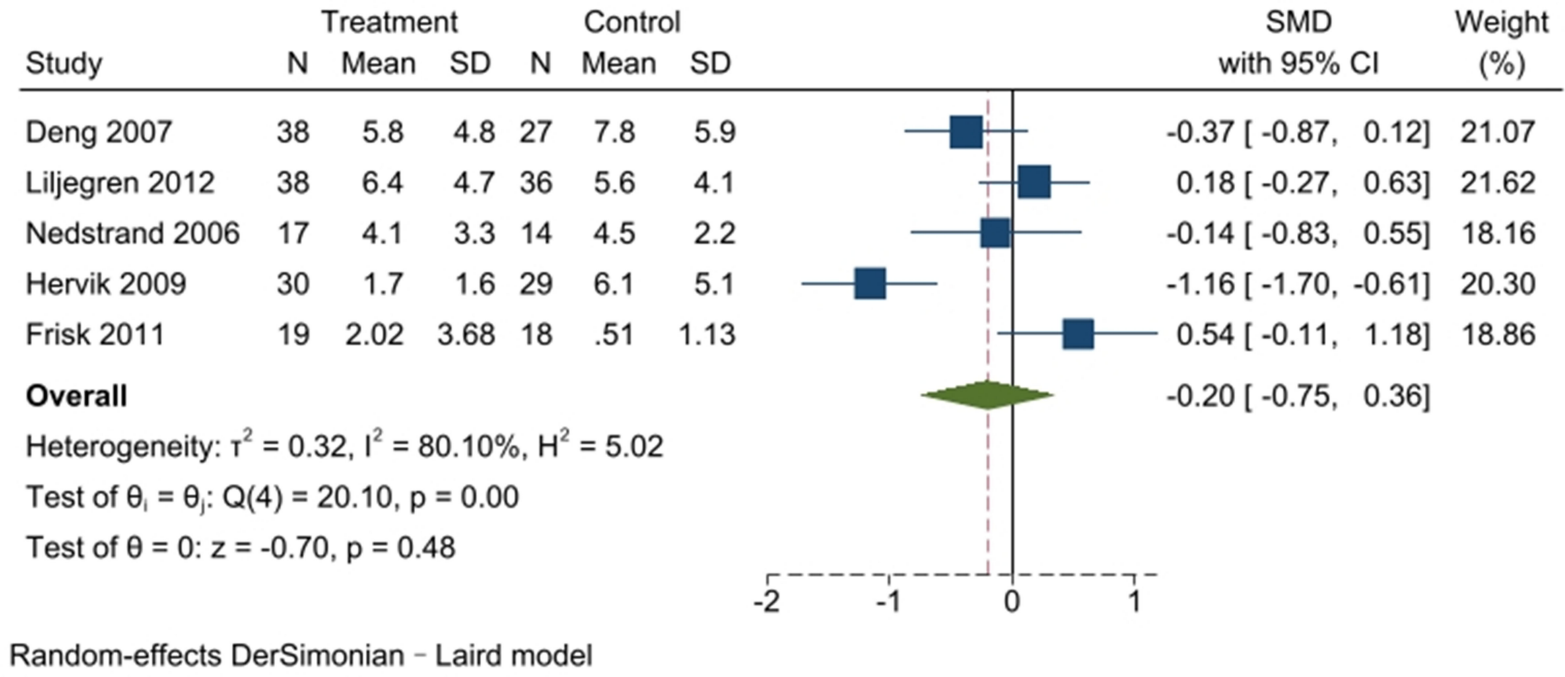
Meta-analysis of acupuncture for the frequency of hot flashes.

The primary outcome indicators of this study encompassed two domains: the frequency of hot flashes and the associated scales for assessing hot flash symptoms. In light of the inclusion of studies featuring diverse measurement indicators, these were transformed into a standardized effect size, represented by SMD ± standard deviation. The meta-analysis of hot-flash frequency incorporated five articles, as shown in **Figure 4**. The Q-test (*I* ^2^ = 80.10%, *P* < 0.001) indicated considerable heterogeneity across the selected studies. Consequently, a random-effects model was used for the meta-analysis. The meta-analysis of all five articles yielded results indicating that the acupuncture group did not exhibit a significant reduction in hot flash frequency compared to the control group (SMD, -0.20; 95% CI, -0.75 to 0.36; *P* = 0.48).

### 3.5 Meta-analysis of hot flash symptoms

**Figure 5:**
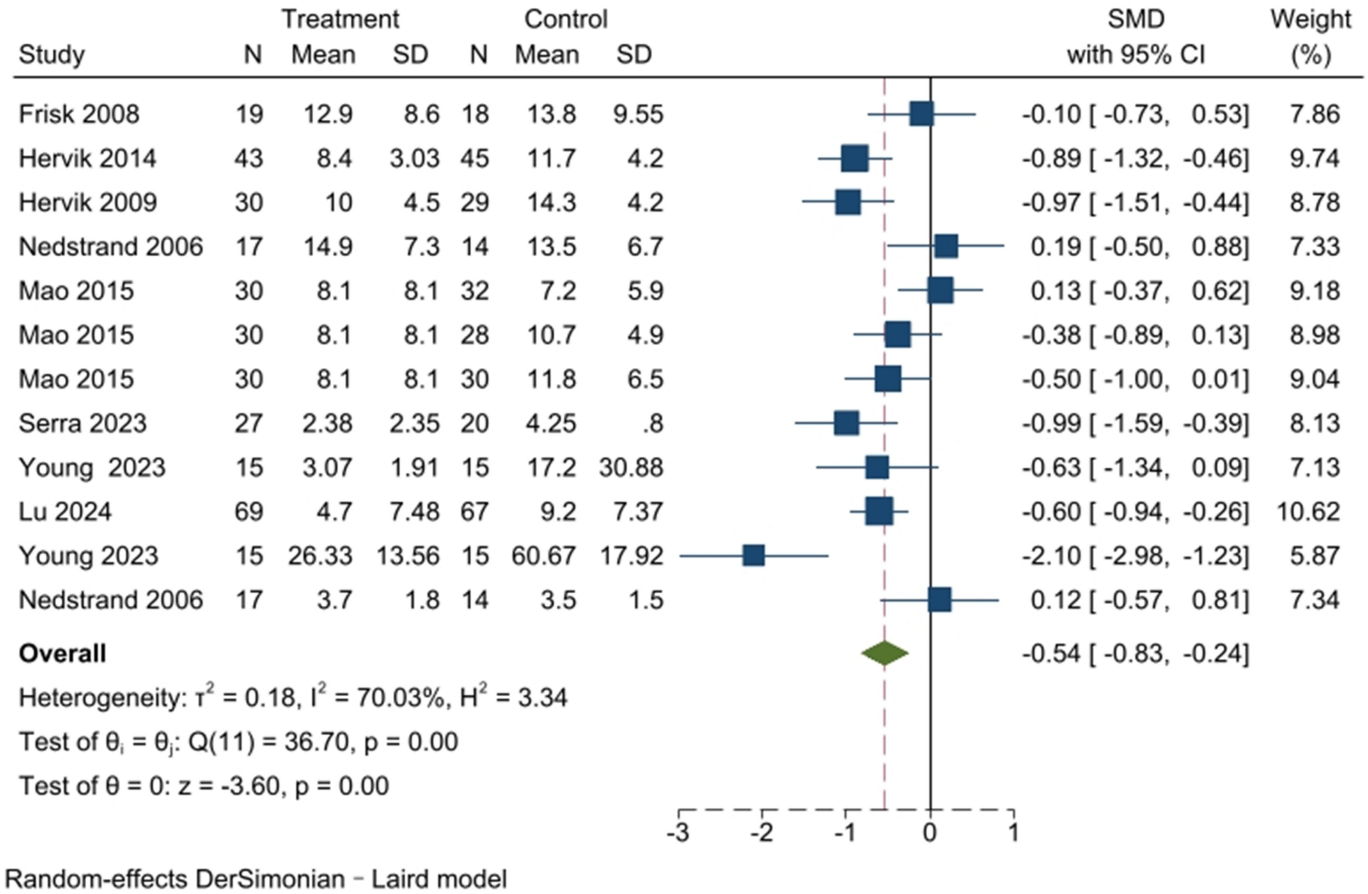
Meta-analysis of acupuncture for hot flash symptoms.

The meta-analysis of scales associated with hot flash symptoms included ten articles, as presented in **Figure 5**. The Q-test (*I* ^2^ = 70.03%, *P* < 0.001) indicated significant heterogeneity among the selected studies. Therefore, a random-effects model was used for meta-analysis. The results indicate that acupuncture can improve hot flash symptoms (SMD, -0.54; 95% CI, -0.83 to -0.24; *P <* 0.05).

### 3.6 Subgroup analysis by frequency of hot flashes

#### 3.6.1 Hot Flashes/24 h *vs*. hot flashes/night

**Figure 6:**
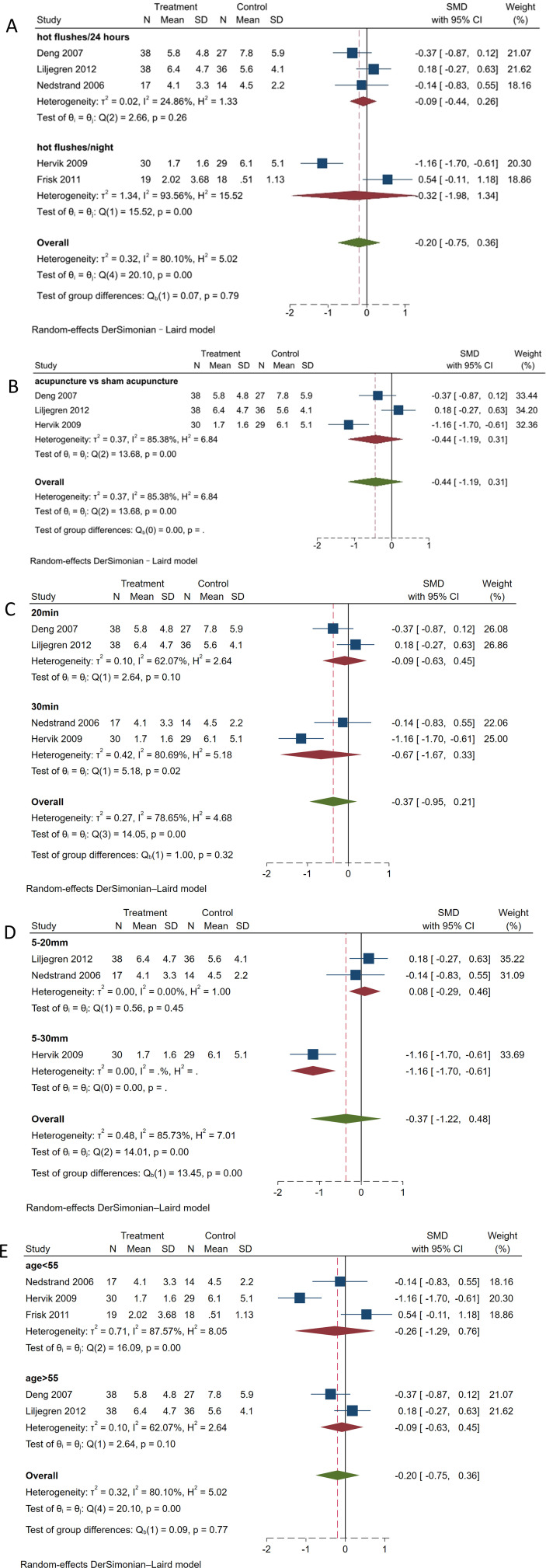
Subgroup analysis of frequency of hot flashes. **(A)** Subgroup analysis by different frequencies. **(B)** Subgroup analysis by different control measures. **(C)** Subgroup analysis by needle retention time. **(D)** Subgroup analysis by needle insertion depth. **(E)** Subgroup analysis by age.

Considering the high heterogeneity in the acupuncture group regarding the frequency of hot flashes and hot flash symptom scores, we conducted a subgroup analysis to explore the sources of this heterogeneity. First, subgroup analysis was performed based on the frequency of hot flashes (**Figure 6A**). Acupuncture in improving the frequency of hot flashes within 24 h (SMD, -0.09; 95% CI, -0.44 to 0.26; *P* = 0.26, *I^2^* = 24.86%) showed a reduction in heterogeneity. In terms of the frequency of hot flashes/night (SMD, -0.32; 95% CI, -1.98 to 1.34; *P* < 0.001, *I* ^2^ = 93.56%), heterogeneity increased compared to before.

#### 3.6.2 Acupuncture vs. sham acupuncture

A subgroup analysis was conducted based on the type of control group (**Figure 6B**). The improvement in hot flash symptoms with acupuncture compared to sham acupuncture was not statistically significant (SMD, -0.44; 95% CI, -1.19 to 0.31; *I^2^* = 85.4%), and there was significant heterogeneity among the studies, which may be due to different study designs and patient populations. Further research is needed to determine the effectiveness of acupuncture, and more rigorous designs and methods should be adopted to reduce heterogeneity.

#### 3.6.3 Needle retention time

Subgroup analysis conducted based on needle retention time (**Figure 6C**) revealed slightly decreased heterogeneity for 20 min of needle retention (SMD, -0.09; 95% CI, -0.63 to 0.45; *P* = 0.1, *I^2^* = 62.07%). For 30 min of needle retention (SMD, -0.67; 95% CI, -1.67 to 0.33; *P* = 0.02, *I^2^* = 80.69%), heterogeneity increased compared to the previous measurement. No statistically significant difference was found between the two groups (*P* = 0.32).

#### 3.6.4 Needle insertion depth

Based on the depth of insertion for subgroup analysis (**Figure 6D**), an insertion depth of 5–20 mm (SMD, 0.08; 95% CI, -0.29 to 0.46; *P* = 0.56, *I²* = 0%) had no heterogeneity. In contrast, an insertion depth of 5–30 mm (SMD, -1.16; 95% CI, -1.70 to -0.61). The difference between the two groups was statistically significant (*P <*0.01). Considering the significant heterogeneity (*I²* = 85.73%), the results should be cautiously interpreted.

#### 3.6.5 Age

Subgroup analysis conducted based on age (**Figure 6E**) demonstrated increased heterogeneity for individuals aged <55 years (SMD, -0.26; 95% CI -1.29 to 0.76; P <0.01, *I²* = 87.57%). For individuals aged >55 years (SMD, -0.09; 95% CI -0.63 to 0.45; *P* = 0.10, *I²* = 62.07%), heterogeneity was lower. No statistically significant difference was found between the two age groups (*P* = 0.77).

### 3.7 Subgroup analysis by the associated scales for assessing hot flash symptoms

#### 3.7.1 Acupuncture *vs*. control group

**Figure 7:**
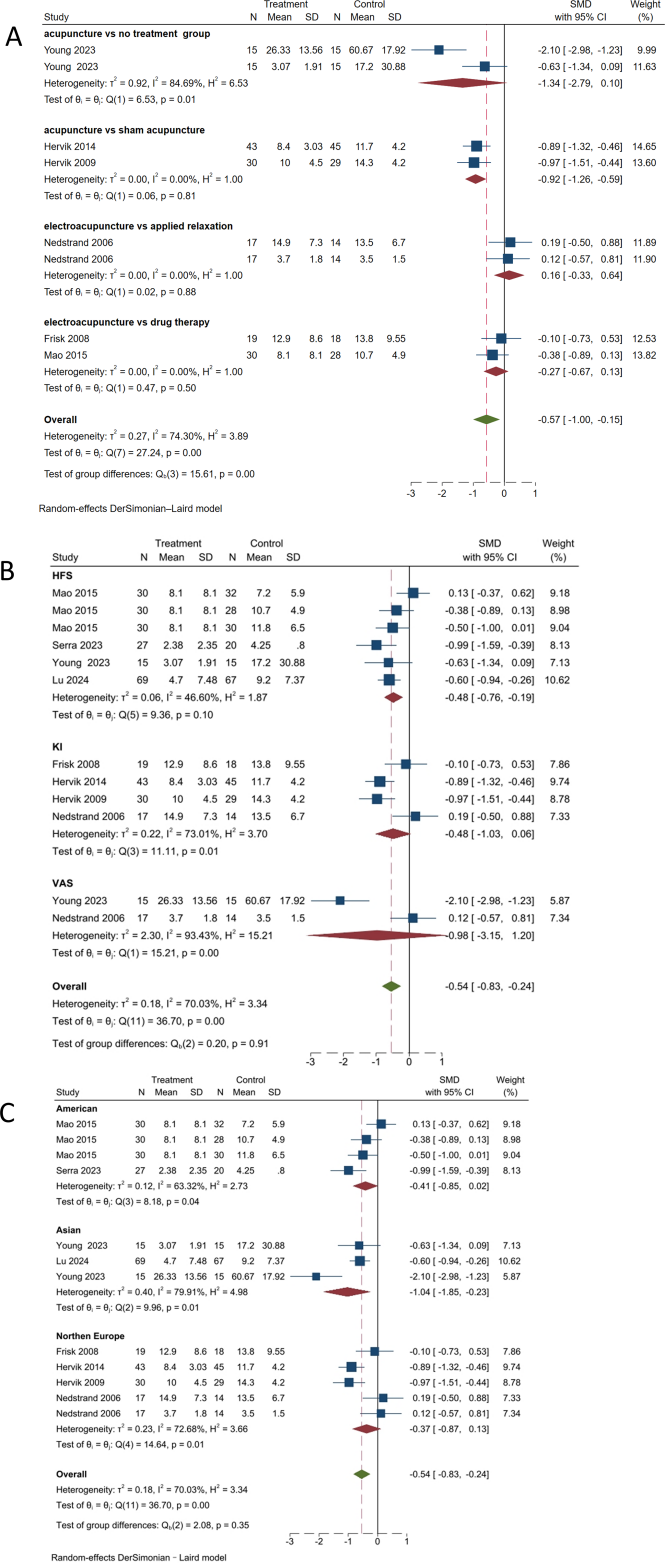
Subgroup analysis of hot flash-related symptom scale. **(A)** Subgroup analysis by different control types. **(B)** Subgroup analysis by different scales. **(C)** Subgroup analysis by different regions.

A subgroup analysis was conducted based on the scales used to assess hot flash symptoms, as shown in **Figure 7A** The statistically significant difference in intra-group heterogeneity between the manual acupuncture and the no treatment group (SMD, -1.34; 95% CI, -2.79 to 0.10; *P* = 0.01, *I*² = 84.69%) highlighted the effect of acupuncture. There was no heterogeneity observed between the acupuncture group and the sham acupuncture group (SMD, -0.92; 95% CI, -1.26 to -0.59; *P =* 0.81, *I*² = 0%), electroacupuncture group and applied relaxation group (SMD, 0.16; 95% CI, -0.33 to 0.64; *P* = 0.88, *I*² = 0%), and the hormone therapy group (SMD, -0.27; 95% CI, -0.67 to 0.13; *P* = 0.50, *I*² = 0%). The overall effect size (SMD, -0.57; 95% CI, -1.00 to -0.15; *P* < 0.001) suggests that while there is some evidence to support the effectiveness of acupuncture, the evidence is not consistent. Further research with stricter experimental designs is required to draw definitive conclusions.

#### 3.7.2 Outcome measures

Given the high possibility of heterogeneity sources caused by different scales, as shown in **Figure 7B**, using the HFS score as the outcome indicator showed a reduction in heterogeneity (SMD, -0.48; 95% CI, -0.76 to -0.19; *P* = 0.10, *I*² = 46.6%). The subgroup analysis with KI score as the outcome indicator showed an increase in heterogeneity (SMD, -0.48; 95% CI, -1.03 to 0.06; *P* = 0.01, *I*² = 73.01%). The subgroup analysis with visual analog scale score as the measure of outcome showed an increase in heterogeneity (SMD, -0.98; 95% CI, -3.15 to 1.20; *P <* 0.001, *I*² = 93.43%). There were statistically significant differences between the KI and visual analog scale groups; however, there was no significant difference in intergroup heterogeneity (*P* = 0.91). Therefore, it is necessary to unify outcome indicators as much as possible.

#### 3.7.3 Region

Considering that current studies have primarily focused on Northern Europe and the United States, with fewer studies from Asia, there is a high possibility of regional differences. As shown in **Figure 7C**, in the United States (SMD, -0.41; 95% CI, -0.85 to 0.02; *P* = 0.04, *I^2^* = 63.32%), heterogeneity has decreased. In Asia (SMD, -1.04; 95% CI, -1.85 to -0.23; *P* = 0.01, *I^2^* = 79.91%), heterogeneity has increased compared to before. In Northern Europe (SMD, -0.37; 95% CI, -0.87 to 0.13; *P* = 0.01, *I^2^* = 72.68%), heterogeneity has increased compared to previously. The differences between the treatment and control groups within each group and in the overall analysis were statistically significant (*P* < 0.05). There was significant heterogeneity among studies from different regions, indicating that the differences between studies may be influenced by various factors such as regional culture and population characteristics, which should be considered in future studies.

#### 3.7.4 Needle retention time

**Figure 8:**
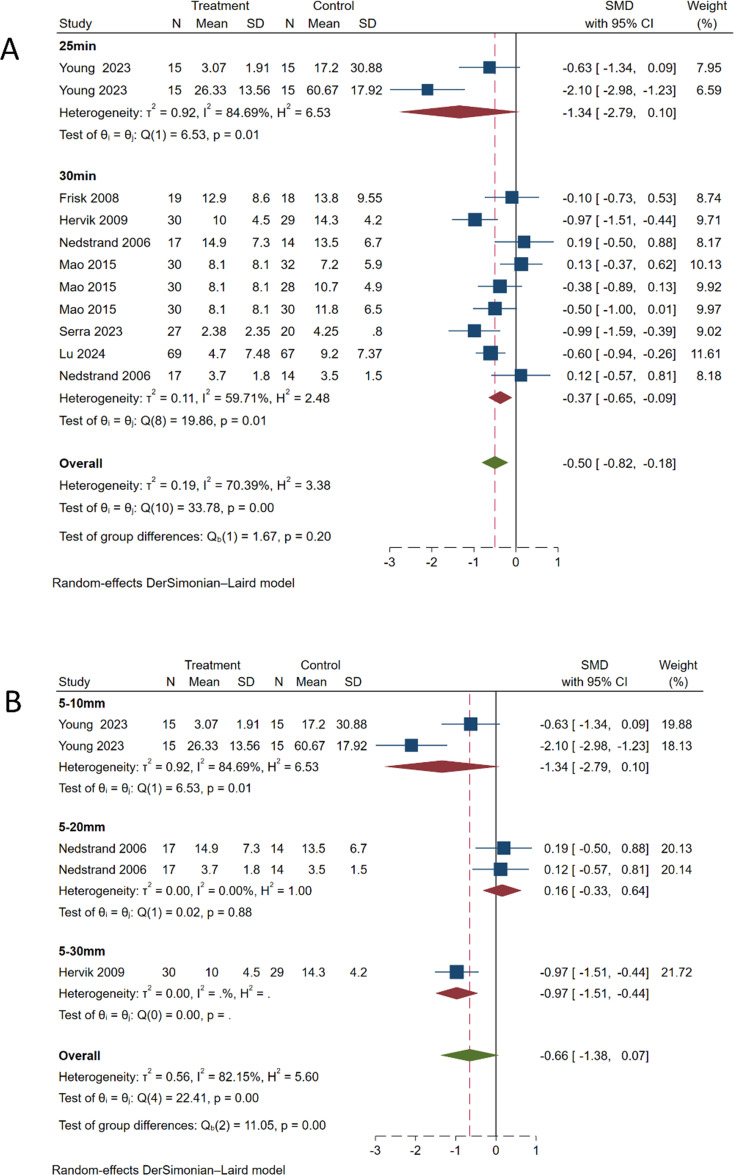
Subgroup analysis of hot flash symptom scale. **(A)** Subgroup analysis by needle retention time. **(B)** Subgroup analysis by needle insertion depth.

Subgroup analysis performed based on needle retention time (**Figure 8A**) showed significant heterogeneity for the 20-min retention group (SMD, -1.34; 95% CI, -2.79 to 0.10; *P* = 0.01, *I^2^* = 84.69%). For the 30-min retention group (SMD, -0.37; 95% CI, -0.65 to -0.09; *P* = 0.01, *I^2^* = 59.71%), the heterogeneity was somewhat reduced. No statistically significant difference was observed between the two groups (*P* = 0.20), indicating that needle retention time has little effect on the therapeutic outcome. However, considering the high heterogeneity (*I^2^* = 70.39%), further analysis focusing on needle retention time should be conducted in the future.

#### 3.7.5 Needle insertion depth

Subgroup analysis conducted based on the depth of needle insertion (**Figure 8B**) revealed significant heterogeneity for the 5–10 mm depth group (SMD, -1.34; 95% CI, -2.79 to 0.10; *P* = 0.01, *I^2^* = 84.69%). For the 5–20 mm depth group (SMD, 0.16; 95% CI, -0.33 to 0.64; *P* = 0.88, *I^2^* = 0%), no significant heterogeneity was observed. For the 5–30 mm depth group (SMD, -0.97; 95% CI, -1.51 to -0.44). The overall meta-analysis pooled effect (SMD, -0.66; 95% CI, -1.38 to 0.07; *P* < 0.01, *I^2^* = 82.15%) indicated significant heterogeneity among different groups.

### 3.8 Meta-regression

Ten studies related to the associated scales for assessing hot flash symptoms were included, and a meta-regression analysis was performed. Given the high suspicion of heterogeneity caused by different scale types, a meta-regression was conducted with effect size as the dependent variable and scale type as the independent variable, yielding a result of *P* = 0.572. Considering that the type of control group might be a source of heterogeneity, the meta-regression results showed *P* = 0.606. Additionally, considering the region as a potential source of heterogeneity, the meta-regression revealed a *P*-value of 0.208. Meta-regression analysis showed *P*=0.257, *P*=0.057, and *P*=0.828 for age, needle retention time, and needle insertion depth, respectively. These results suggest that they were not sources of heterogeneity. Considering the high heterogeneity in this study, the results of the meta-regression may not be reliable; therefore, a comprehensive evaluation should be conducted in conjunction with subgroup and sensitivity analyses.

### 3.9 Sensitivity analysis

**Figure 9:**
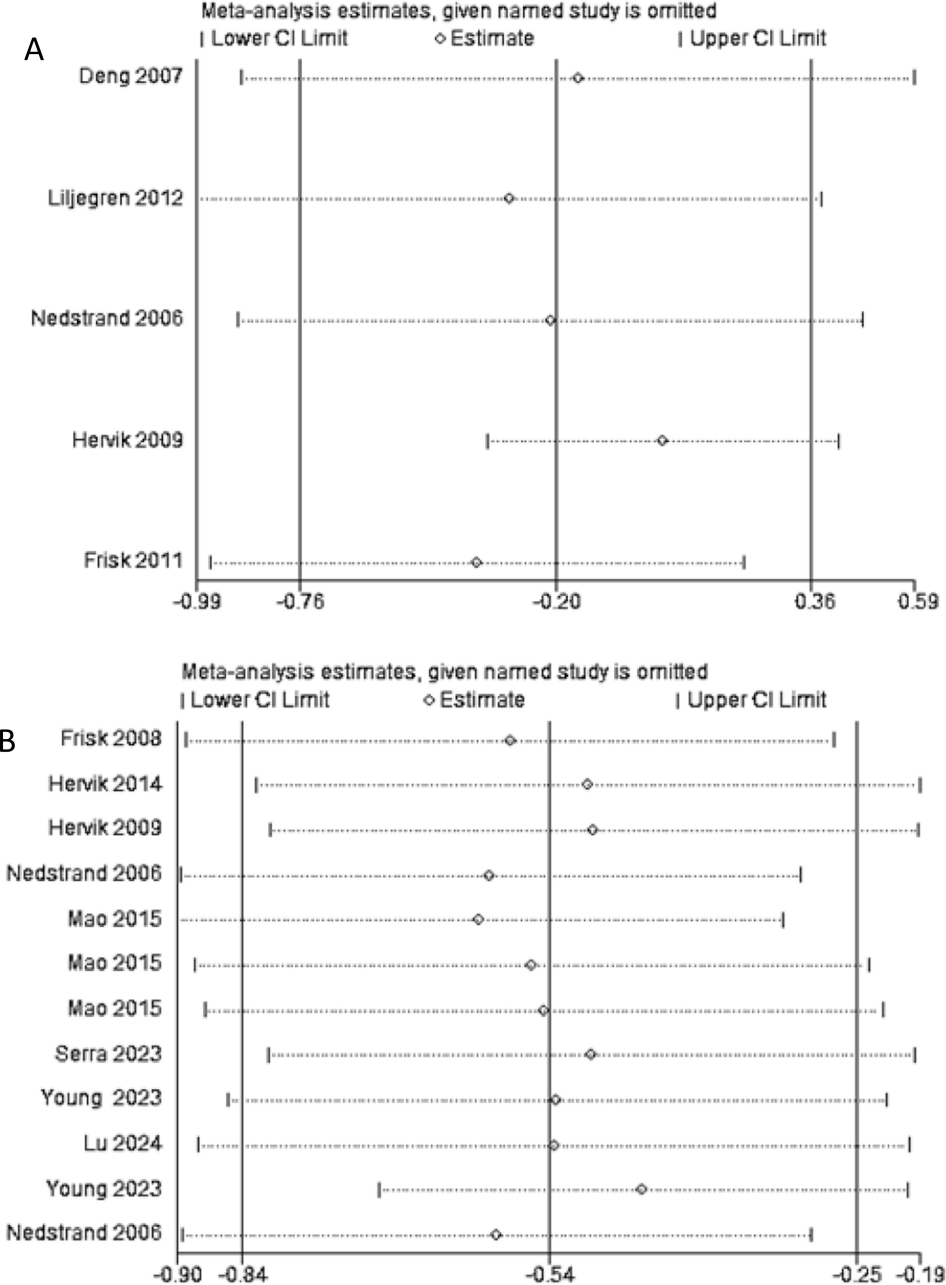
**(A)** Sensitivity analysis of frequency of hot flashes; **(B)** Sensitivity analysis of hot flash-related symptom scale.

Sequential exclusion of each of the 11 included studies was conducted, followed by recalculation of the meta-analysis results following each exclusion. As shown in **Figures 9A, B**, the recalculated summary estimates remained largely unchanged after the sequential exclusion of individual studies. This suggests that the findings of the meta-analysis within this study are robust and dependable.

### 3.10 Publication bias

**Figure 10:**
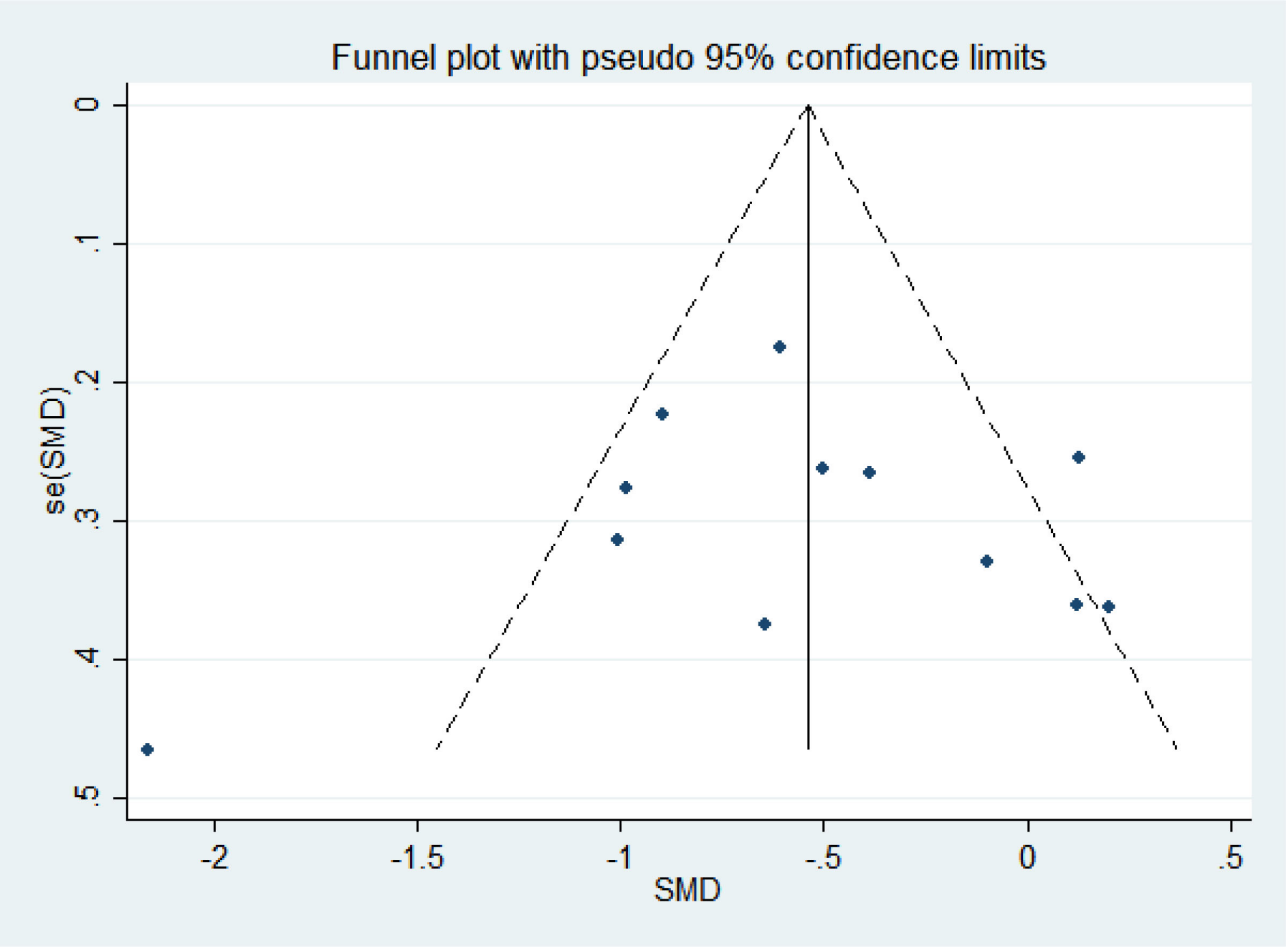
The funnel plot related to hot flash-related symptom scale.

As shown in **Figure 10**, the scatter distribution was uniform and essentially symmetrical on both the left and right sides, indicating no significant publication bias. Publication bias was assessed using the Begg and Egger tests. The Begg test result was *P* = 0.837 and the Egger test result was *P* = 0.903, both suggesting that there was no publication bias.

### 3.11 Quality of evidence

Using GERAD pro GDT software to evaluate different outcome indicators, it was found that the evidence provided by Frisk et al. (30), Hervik et al. (25), and Nedstrand, et al. (27) in terms of the KI index was rated as “very low,” and the evidence provided by Hervik et al. (24) on the KI index was rated as “low.” The evidence provided by Deng et al. (23) and Liljegren et al. (28) for the frequency of hot flashes per 24 h was rated as “very low.” In terms of the night-time hot flash frequency, the evidence provided by Frisk et al. (29) was rated as “very low” and the evidence provided by Hervik et al. (24) was rated as “low.” In terms of the hot flash score, two of the articles (14, 22) had “very low” evidence and two had “low” evidence (20, 32). In terms of the visual analog scale, one article (27) had evidence rated as “very low” and another (22) had evidence rated as “very low.” For further details, refer to **Table 3**.

**Table 3:** GRADE evidence profile in the meta-analysis.

Certainty assessment							№ of patients		Certainty	Importance
№ of studies	Study design	Risk of bias	Inconsistency	Indirectness	Imprecision	Other considerations	Acupuncture	Conctrol group		
Kupperman’s Index										
Frisk 2008 (30)	randomised trials	not serious	serious^a^	serious^b^	serious^b^	none	19	18	⊕⚪⚪⚪ Very low^a,b^	CRITICAL
Hervik 2014 (25)	randomised trials	very serious^a^	very serious^a^	serious^c^	serious^d^	none	43	45	⊕⚪⚪⚪ Very low^a,c,d^	CRITICAL
Jill 2009 (24)	randomised trials	not serious	not serious	serious^d^	serious^c^	none	30	29	⊕⊕⚪⚪ Low^c,d^	CRITICAL
Nedstrand 2006 (27)	randomised trials	not serious	not serious	very serious^b^	serious^d^	none	17	14	⊕⚪⚪⚪ Very low^b,d^	CRITICAL
hot flushes/24 hours										
Deng 2007 (23)	randomised trials	not serious	serious^b^	not serious	very serious^c^	none	38	27	⊕⚪⚪⚪ Very low^b,c^	CRITICAL
Liljegren 2012 (28)	randomised trials	very serious^a^	very serious^b^	very serious^c^	very serious^d^	none	38	36	⊕⚪⚪⚪ Very low^a,b,c^	CRITICAL
hot flushes/night										
Frisk 2011 (29)	randomised trials	very serious^a^	very serious^b^	very serious^c^	very serious^d^	none	19	18	⊕⚪⚪⚪ Very low^a,b,c,d^	CRITICAL
Jill 2009 (24)	randomised trials	not serious	not serious	serious^d^	serious^c^	none	30	29	⊕⊕⚪⚪ Low^c,d^	CRITICAL
Hot Flash Score										
Mao 2015 (20)	randomised trials	not serious	not serious	serious^c^	serious^d^	none	30	30	⊕⊕⚪⚪ Low^c,d^	CRITICAL
Serra 2023 (22)	randomised trials	very serious^a^	very serious^a^	very serious^c^	very serious^d^	none	27	20	⊕⚪⚪⚪ Very low^a,c,d^	CRITICAL
Young 2023 (32)	randomised trials	not serious	not serious	serious^c^	serious^d^	none	15	15	⊕⊕⚪⚪ Low^c,d^	CRITICAL
Lu 2024 (14)	randomised trials	very serious^a^	very serious^c^	serious^d^	serious^d^	none	69	67	⊕⚪⚪⚪ Very low^a,c,d^	CRITICAL
Visual Analogue Scale										
Young 2023 (32)	randomised trials	not serious	not serious	serious^c^	serious^d^	none	15	15	⊕⊕⚪⚪ Low^c,d^	CRITICAL
Nedstrand 2006 (27)	randomised trials	not serious	not serious	very serious^b^	serious^d^	none	17	14	⊕⚪⚪⚪ Very low^b,d^	CRITICAL

a. The included studies have a large bias in methodology such as randomization, allocation concealment, and blinding.

b. The confidence interval overlaps less or the I2 value of the combined results was larger.

c. The sample size from the included studies does not meet the optimal sample size or the confidence interval was narrow enough.

d. The inclusion of a small number of patients, short observation time, and wide confidence intervals resulted in a decrease in reliability.

### 3.12 Adverse events

Adverse events were documented in two studies. One study revealed that the acupuncture group encountered minimal side effects, including slight bleeding or bruising at the insertion site that did not require additional medical treatment. There were a total of 14 grade 1 adverse events recorded among 12 participants (23). Another study indicated that only four women in the acupuncture group had mild and transient side effects, whereas 14 (15%) in the medication group reported symptoms of fatigue, itching, and nausea (19).

## 4 Discussion

Our meta-analysis results show that acupuncture does not have a significant advantage in reducing the frequency of hot flash (SMD, -0.20; 95% CI, -0.75 to 0.36; *P* = 0.48, *I^2^* = 80.19%); however, in terms of hot flash scores, acupuncture has a certain therapeutic effect compared with sham acupuncture (SMD, -0.92; 95% CI, -1.26 to -0.59; *P*<0.01, *I^2^* = 0%), which is consistent with the results of a network meta-analysis published in 2020 (33), reflecting that acupuncture has a certain efficacy in improving hot flash symptoms. Nevertheless, a meta-analysis from 2016 demonstrated that acupuncture has no significant advantage in reducing the frequency and severity of hot flashes (*P* = 0.34 and *P* = 0.33, respectively), which contradicts our results (34). This discrepancy may be due to our study including more and the latest randomized controlled trials, which also indirectly demonstrates that acupuncture continues to show potential in treating breast cancer-related hot flashes. Therefore, high-quality clinical research is needed to further clarify whether acupuncture should be strongly recommended as a routine adjunctive therapy for breast cancer patients.

Heterogeneity was a major challenge in our study and we considered those related to the outcome measures, types of controls, regional differences, and other factors. Firstly, the outcome measures for breast cancer-related hot flashes are diverse, and there is currently no consensus on a unified evaluation standard, which is one of the reasons for the significant heterogeneity observed among different studies. Therefore, we call for the establishment of unified outcome measures in the future to more accurately assess the true efficacy of acupuncture. Second, the diversity of control groups is also a potential source of heterogeneity. The control groups in this study include various drug treatments, sham acupuncture, and others. Sham acupuncture is a significant issue in acupuncture clinical trials, with some researchers believing that despite the use of sham acupuncture, the non-specific effects of acupuncture have not been completely excluded, leading to the view that acupuncture is a “powerful placebo.” Research has shown that through functional magnetic resonance imaging (fMRI), it has been found that sham acupuncture can stimulate the anterior cingulate cortex and thalamus, potentially mimicking the effects of real acupuncture (35). Therefore, considering the limitations of sham acupuncture, we believe that future research should further optimize its design and establish a no-treatment control group for comparison to distinguish its specific effects and increase the homogeneity of the studies. Additionally, current clinical research on acupuncture treatment for hot flashes in breast cancer patients lacks comparisons based on the subdivision of drug types. The maximum effect (Emax) pharmacokinetic model for venlafaxine is 13.9%, and for gabapentin, it is 14.8% (36). Therefore, future research can establish different drug control groups to compare the efficacy between specific drug groups and acupuncture groups. Third, the heterogeneity caused by regional differences must be considered. We conducted subgroup analysis by region but did not find the source of heterogeneity as we expected. Currently, most acupuncture research focuses on populations in the United States and Europe, with only a few studies on acupuncture in Asian patients with breast cancer (26, 37). Research shows that black and Hispanic people report a higher incidence of hot flashes and more severe endocrine symptoms (38). A multinational trial by Lu et al. (14) found that a 10-week acupuncture intervention significantly reduced endocrine symptoms and hot flashes in the United States, China, and South Korea. There were differences in efficacy across the centers, with the most significant impact in South Korea and the least in China. Therefore, biological and sociocultural differences among regional populations may lead to heterogeneity. Unfortunately, due to the lack of detailed data in current research, we are unable to conduct further in-depth exploration. Fourth, Huangdi Neijing (The Yellow Emperor’s Classic of Medicine) was the first to mention the theory of constitutional types. Chinese Traditional Chinese Medicine physicians have extended this theory and proposed a distinction between constitutional and syndrome patterns to advance the application of differential diagnosis and treatment based on the constitution, disease, and syndrome, highlighting the importance of personalized medicine. The constitution is a crucial factor in the formation of syndrome patterns and directly influences the clinical manifestations of the disease. Differences in the constitution determine the occurrence, pathogenesis, and prognosis of various diseases (39, 40). Traditional Chinese medicine considers the nature of breast cancer to be rooted in its deficiency, which manifests in excess. This deficiency is mainly characterized by disorders of the Chong and Ren meridians and deficiency of the liver and kidney, whereas excess is predominantly manifested as qi stagnation, phlegm turbidity, and blood stasis. Chinese experts in traditional Chinese medicine generally classify breast cancer into the following syndrome patterns: qi and blood deficiency, Chong and Ren disorders, liver depression and phlegm coagulation, toxin accumulation, spleen deficiency and phlegm dampness, and liver and kidney yin deficiencies. Among the patients with breast cancer, the proportions of patients with yang deficiency, qi stagnation, qi deficiency, yin deficiency, and balanced constitutions were relatively high. Qi stagnation is often related to liver depression and phlegm coagulation; qi deficiency is frequently associated with qi and blood deficiency; and yin deficiency is linked to liver and kidney yin deficiency (41). These correlations indicate that there is a relationship between the TCM constitution and syndrome classification in breast cancer, which influences the occurrence, development, and subsequent treatment of the disease to some extent. Additionally, there is a connection between the clinical and pathological characteristics of breast cancer and TCM constitution. Yin deficiency is related to tissue grading, with a higher grade increasing the likelihood of its occurrence; qi deficiency is associated with pathological types; phlegm dampness is related to lymph node metastasis, with a higher number of metastases increasing its likelihood; and a balanced constitution is related to tissue grading and hormone receptor expression, with a higher grade increasing its likelihood (42, 43). However, current evidence-based medical research on acupuncture treatment for hot flashes in breast cancer rarely includes the constitution or syndrome patterns. Therefore, this may be one of the reasons for the heterogeneity. Overall, Traditional Chinese medicine emphasizes the differential treatment of individualized syndrome patterns from a macro perspective (44). Syndrome differentiation and treatment are core concepts of TCM. We advocate that Chinese scholars conduct evidence-based clinical research, incorporating the constitution and syndrome patterns into the analysis to achieve more accurate personalized treatment from a TCM perspective. We also suggest using modern scientific research methods, such as epidemiological surveys, genomics, and molecular biology, to systematically investigate the relationship between the TCM constitution, syndrome patterns, and heterogeneity. Fifth, we conducted subgroup analyses on needle retention time, needle depth, and patient age, and meta-regression showed that these factors did not account for the source of heterogeneity. At the same time, We also consider that the source of heterogeneity may be related to different breast cancer subtypes and stages. Unfortunately, the studies included in this research did not specify breast cancer subtypes and stages in detail. Additionally, the efficacy of acupuncture is influenced by various factors, including the type of needle, acupoint selection, treatment course and frequency, and technical level of the practitioner, all of which may contribute to the observed heterogeneity (45). Although this study explored the sources of heterogeneity, it could not fully explain them. Through sensitivity analysis, we confirmed the stability of the results, indicating that our conclusions are relatively reliable. The Egger test and funnel plot showed no evidence of publication bias. The quality of evidence was low, with most studies having a high risk of bias, lacking concealment of allocation and blinding, which may have contributed to significant methodological heterogeneity. Therefore, it is necessary to optimize trial design, control variables, and conduct high-quality clinical trials to confirm the efficacy of acupuncture in treating cancer-related hot flashes.

### 4.1 Implications for future research

**Figure 11:**
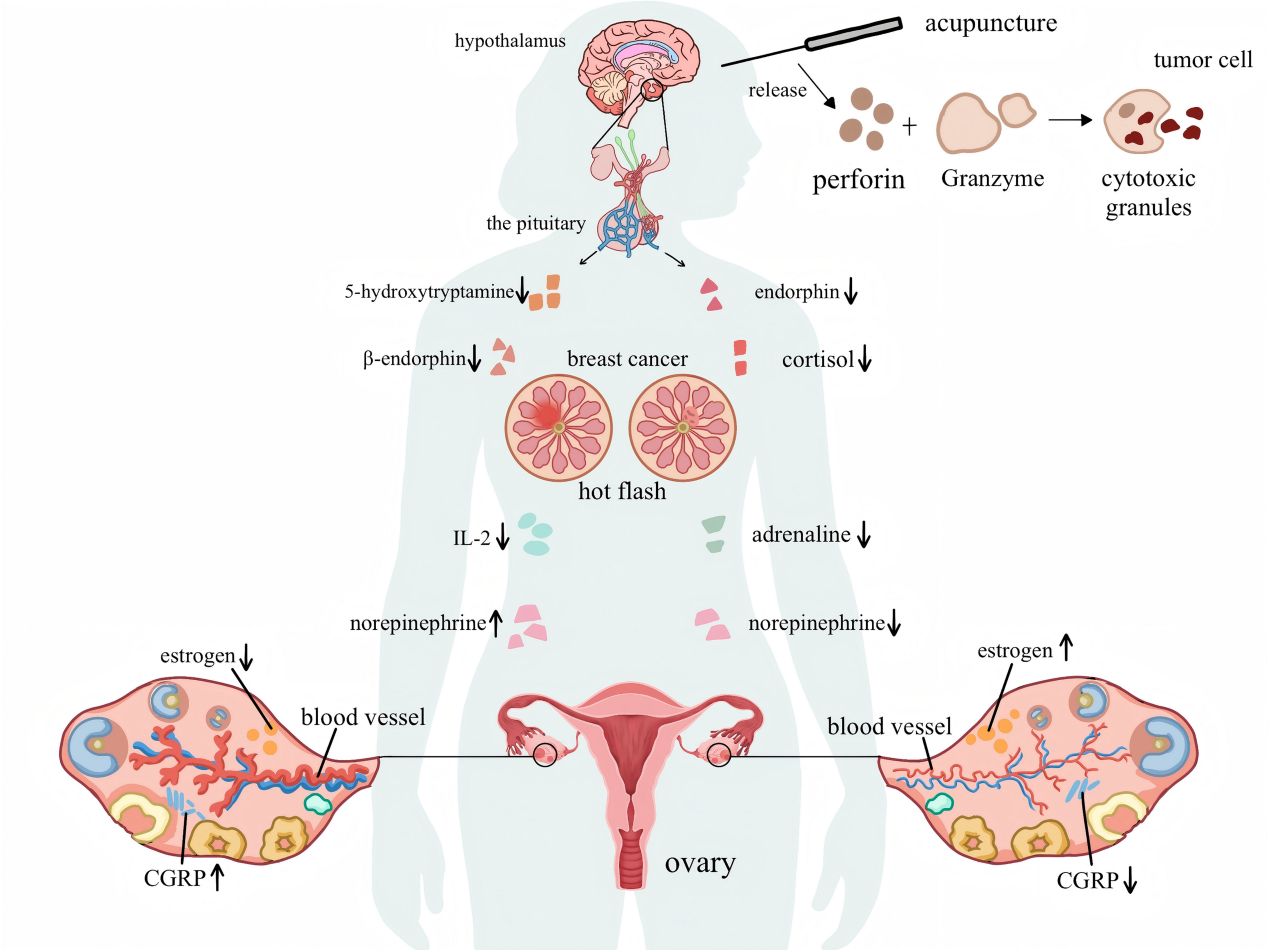
The mechanism diagram of breast cancer hot flashes and the mechanism of acupuncture in improving hot flashes.

Acupuncture, as an adjuvant therapy for alleviating cancer-related symptoms, is both safe and effective (46). In oncology, acupuncture has been incorporated into clinical practice guidelines for managing symptoms such as breast cancer-related hot flashes, highlighting its potential and prospects in treating cancer-related symptoms (47). However, a significant disparity exists in the quality and level of evidence-based data available for acupuncture in treating hot flashes in patients with breast cancer, highlighting the importance of further exploring its mechanism. As shown in **Figure 11**, some studies have suggested that the occurrence of hot flashes is related to a decrease in estrogen levels, which is associated with various factors. This decline may cause a series of changes in neural and humoral factors, indirectly leading to hot flash symptoms (48). There are several views on this, the first of which is the hypothalamic-pituitary-ovarian axis theory. The chemotherapy drugs and radiotherapy factors for patients with breast cancer may lead to premature ovarian failure (49), resulting in the disappearance of the feedback inhibition mechanism on the pituitary gland; this imbalance affects the hypothalamic-pituitary-ovarian axis, causing hyperfunction of the hypothalamus and pituitary gland (50). The second is the endocrine-neuroregulatory mechanism. By reducing estrogen levels or blocking its receptor action to inhibit cancer cell growth, the decrease in blood estrogen directly affects the central nervous system. It suppresses the production of neurotransmitters such as 5-hydroxytryptamine and β-endorphin, both of which can regulate the hypothalamic temperature set point and inhibit norepinephrine secretion. When their levels decrease, it directly lowers the temperature set point, leading to dysfunction of the hypothalamic thermoregulatory center. At the same time, it eliminates the negative feedback inhibition on norepinephrine, and the dual action leads to hot flashes (51). Third, reduced estrogen levels decrease the expression of receptors on the surface of immune cells, resulting in insufficient synthesis of immune mediators such as interleukin-2. This immune imbalance results in abnormal levels of inflammatory cytokines, which in turn affect neurotransmitter function, prompting excessive release of norepinephrine and causing hot flashes (52). Fourth, research has indicated that a decrease in estrogen levels stimulates the release of calcitonin gene-related peptide from perivascular calcitonin gene-related peptide fibers, leading to the disruption of vascular homeostasis and vasodilation, which triggers hot flashes (53).

The mechanism by which acupuncture improves hot flashes remains inconclusive; however, it can be generally categorized into the following aspects: First, by regulating estrogen levels, studies have shown that acupuncture at acupoints such as Guanyuan (CV 4), Neiguan (PC 6), and Zusanli (ST 36) can increase the serum estradiol levels and hypothalamic 5-hydroxytryptamine content in ovariectomized rats (54), reduce the levels of follicle-stimulating and luteinizing hormones, effectively regulate the hypothalamic-pituitary-ovarian axis function, maintain the homeostasis of the thermoregulatory center, and consequently reduce hot flashes. Second, acupuncture may influence neurotransmitter activities by regulating endorphins and norepinephrine in the central nervous system. Acupuncture can reduce the level of substance P and enhance the release of endogenous opioid peptides (55–57). Research has found that electroacupuncture stimulation of the Zusanli (ST36) and ST25 points in mice can activate the sympathetic nerves and adrenal medulla, promoting the release of norepinephrine (58, 59) and improving hot flashes by affecting neurotransmitters. Third, electroacupuncture can regulate vascular relaxation factors by downregulating the expression of calcitonin gene-related peptides (60, 61), thereby reducing the vasodilatory response to alleviate hot flashes. Fourth, research has shown that psychological factors considerably impact the immune system of patients with breast cancer. The mechanism is as follows: chronic stress increases endocrine regulators and cytokines, activating the hypothalamic-pituitary-adrenal axis and the sympathetic nervous system (62); this leads to an increase in the secretion of hormones such as cortisol and adrenaline, which alters the tumor microenvironment and promotes its growth. Meanwhile, chronic stress inhibits the activity of natural killer cells, reducing the body’s ability to kill tumor cells (63). This disruption affects the basic physiological processes of the endocrine and immune systems in patients with breast cancer to regulate tumor growth (64). Simultaneously, acupuncture can enhance the activity and number of natural killer cells, release cytotoxic granules containing perforin and granzymes, improve the body’s ability to kill tumor cells, and alleviate hot flash symptoms by improving the immune status of patients with breast cancer (65–67). As cancer treatment shifts towards personalized treatment, mental health assessments and psychological intervention therapies can be utilized to prevent the occurrence and development of cancer. Further research on the combination of acupuncture and psychological interventions could provide a broader perspective on the relative effectiveness of complementary treatments in maximizing patient benefits.

Acupuncture may also exert its effects by influencing the expression of specific genes. Romero et al. (68) have highlighted that the *ADORA1*, *COMT*, *TCL1A*, and *TRPV1* genes can predict breast cancer survivors’ response to acupuncture for hot flashes. Research has also demonstrated that different molecular subtypes of breast cancer have distinct pathological characteristics, exhibit survival differences, and vary in their sensitivity to different treatment regimens (69). Therefore, future research should explore gene expression regulation as a new direction, perform genetic typing of breast cancer, and identify breast cancer survivors who are more sensitive to acupuncture treatment, thereby achieving personalized treatment regimens, optimizing the management of hot flashes, and maximizing the effects of acupuncture.

To explore the mechanism of acupuncture in greater depth and ensure the reliability and validity of research findings, it is necessary to adopt high-quality control groups in acupuncture research. However, the use of high-quality control groups in acupuncture research poses significant challenges. Research suggests that the type of acupuncture control used, whether at non-acupoint locations or non-penetrating needles, may have specific effects (70, 71). Sham or placebo acupuncture can induce tactile stimulation, activate the somatosensory system, and engage the related brain regions, leading to specific physiological responses (72). In the studies included in this review, Liljegren et al. (28) performed sham needling, in which the needle set was placed 1 cm away from the acupuncture point, without piercing the skin, and rotated to make the patient’s skin sensory. The results showed no significant difference between this sham acupuncture and real acupuncture in terms of improving hot flashes. Therefore, reducing the placebo effect of sham acupuncture is crucial for accurately evaluating the actual effects of acupuncture. Overall, future research should conduct large-scale, multicenter, randomized controlled trials to enhance the generalizability and credibility of the results. The study design should be optimized with the selection of appropriate control groups and consideration of the gold standard for double-blind randomized controlled trials. Emphasis should be placed on improving research quality, including the implementation of randomization, blinding, and allocation concealment, to enhance the reliability and comparability of studies. Standardized acupuncture protocols for hot flashes in breast cancer could be formulated, including the combination of specific acupuncture points, the depth of needling, and the duration of needle retention to minimize the influence of the differences in the treatment protocols on the results of the study. To delve deeper into the mechanisms of acupuncture, functional magnetic resonance imaging (fMRI) techniques can be used to observe the effects of acupuncture on specific areas of the brain, such as the hypothalamus and amygdala, and the relationship between these areas and the occurrence of hot flashes, as well as exploring the regulation of gene expression, genotyping breast cancer, and identifying patients with the type of breast cancer that is more sensitive to acupuncture treatment to optimize hot flash management.

### 4.2 Innovations and limitations

Concerning innovation, this study thoroughly explores and summarizes the mechanisms of acupuncture in treating hot flashes in patients with breast cancer, providing a reference and new directions for future research, thereby offering more robust evidence support for the application of acupuncture in this field. Additionally, compared to previous meta-analyses, this study provides an update, expanding the clinical protocols for acupuncture treatment of hot flashes in patients with breast cancer and enhancing the quality of clinical evidence. Regarding limitations, a high degree of heterogeneity exists among these studies, and the sample sizes are relatively small, which restricts our ability to accurately assess the true efficacy of acupuncture in treating breast cancer-related hot flashes. The overall quality of the research evidence is low, and the subjective nature of the outcome measures may have affected result reliability.

## 5 Conclusion

This meta-analysis revealed that acupuncture did not significantly reduce the frequency of hot flashes in patients with breast cancer. Nevertheless, an analysis of clinical hot flash rating scales indicated that acupuncture can alleviate hot flash symptoms. These findings suggest that the clinical effectiveness of acupuncture for treating hot flashes in patients with breast cancer is characterized by certain uncertainties and limitations. This uncertainty and limitation may be due to the relatively low quality of clinical research in this field, as well as the lack of standardized diagnostic and treatment protocols. To establish definitive evidence of acupuncture’s efficacy in this context, future research should prioritize conducting high-quality, large-scale, multi-center, randomized controlled trials. Additionally, efforts should be made to enhance the rigor of trial methodologies and develop standardized acupuncture protocols to ensure diagnostic and treatment standardization.

## Data Availability

The original contributions presented in the study are included in the article/supplementary material. Further inquiries can be directed to the corresponding author.
